# 

**Published:** 1868-04

**Authors:** 


Vol. V. *January—April*, 1868. Nos. 2 & 3.

### THE IOWA MEDICAL JOURNAL,

EDITED BY

J. C. HUGHES, M. D.,

*Professor of the Institutes, and Practice of Surgery and Surgical Clinics in the Medical Department Iowa State University; Surgeon-in-Charge of the College Infirmary and Eye and Ear Institute; Member of American Medical Association and of the British Association for the Promotion of Science, etc., etc.*

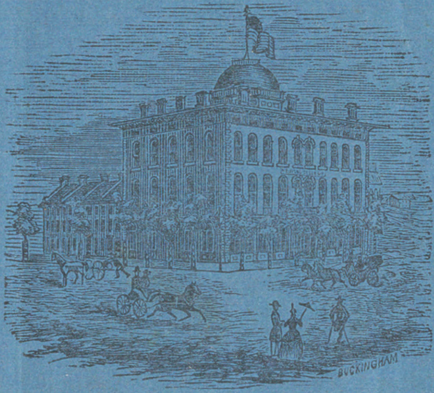

KEOKUK, IOWA.

Editors and Publishers Please Exchange.

Address all Communications, Periodicals or Books to the Editor.

*KEOKUK, IOWA:*

PRINTED AT THE GATE CITY BOOK AND JOB ROOMS.

1868.

**Letters requiring an answer, must contain a Stamp to insure attention.**

